# Recent Advances in Cellular Signaling Interplay between Redox Metabolism and Autophagy Modulation in Cancer: An Overview of Molecular Mechanisms and Therapeutic Interventions

**DOI:** 10.3390/antiox12020428

**Published:** 2023-02-09

**Authors:** Md. Ataur Rahman, Kazi Rejvee Ahmed, Farzana Haque, Moon Nyeo Park, Bonglee Kim

**Affiliations:** 1 Department of Pathology, College of Korean Medicine, Kyung Hee University, Hoegidong Dongdaemungu, Seoul 02447, Republic of Korea; 2 Korean Medicine-Based Drug Repositioning Cancer Research Center, College of Korean Medicine, Kyung Hee University, Seoul 02447, Republic of Korea; 3 Department of Biotechnology and Genetic Engineering, Faculty of Biological Sciences, Islamic University, Kushtia 7003, Bangladesh

**Keywords:** autophagy, cancer, autophagosomes, redox metabolism, redox homeostasis, reactive oxygen species

## Abstract

Autophagy is a fundamental homeostatic process in which certain cellular components are ingested by double-membrane autophagosomes and then degraded to create energy or to maintain cellular homeostasis and survival. It is typically observed in nutrient-deprived cells as a survival mechanism. However, it has also been identified as a crucial process in maintaining cellular homeostasis and disease progression. Normal cellular metabolism produces reactive oxygen (ROS) and nitrogen species at low levels. However, increased production causes oxidative stress, which can lead to diabetes, cardiovascular diseases, neurological disorders, and cancer. It was recently shown that maintaining redox equilibrium via autophagy is critical for cellular responses to oxidative stress. However, little is understood about the molecular cancer processes that connect to the control of autophagy. In cancer cells, oncogenic mutations, carcinogens, and metabolic reprogramming cause increased ROS generation and oxidative stress. Recent studies have suggested that increased ROS generation activates survival pathways that promote cancer development and metastasis. Moreover, the relationship between metabolic programming and ROS in cancer cells is involved in redox homeostasis and the malignant phenotype. Currently, while the signaling events governing autophagy and how redox homeostasis affects signaling cascades are well understood, very little is known about molecular events related to autophagy. In this review, we focus on current knowledge about autophagy modulation and the role of redox metabolism to further the knowledge of oxidative stress and disease progression in cancer regulation. Therefore, this review focuses on understanding how oxidation/reduction events fine-tune autophagy to help understand how oxidative stress and autophagy govern cancer, either as processes leading to cell death or as survival strategies for maintaining redox homeostasis in cancer.

## 1. Introduction

Recent findings suggest that autophagy, which maintains cellular homeostasis and is involved in neurodegeneration and cancer, is responsible for recycling misfolded proteins and damaged organelles. It comprises a series of processes that are meticulously monitored and managed (initiation, nucleation, elongation, lysosomal fusion, and destruction) [1]. It was recently found that altering autophagic activity by targeting particular regulatory actors could change disease processes [2]. Autophagy is an important evolutionary catabolic process involving the digestion of cytoplasmatic components [3]. Autophagy is a persistent homeostatic system, and nearly all cell types have some basal level of autophagy activity [4,5]. In most situations, stress-induced autophagy is a pro-survival process, whereas relatively few examples show autophagy mediating cell death [6]. Therefore, understanding the processes responsible for cancer regulation through oxidative stress and autophagy in redox homeostasis in cancer cells is essential.

Redox metabolism affects cancer initiation, metastasis, proliferation, apoptosis, the tumor microenvironment, metabolic reprogramming, therapeutic resistance, and autophagy [7]. An appropriate reactive oxygen species (ROS) concentration drives carcinogenesis and supports cancer cell development, whereas an excess causes cell death. Cancer cell antioxidant systems reduce tumor-promoting ROS generation [8]. Redox conditions cause tumors. Cancer cells have deregulated ROS generation and limiting mechanisms, influencing cell behavior from signaling to death [9]. ROS modify the tumor environment, impacting the stromal cells that provide metabolic support, blood supply, and immunological responses [10]. While ROS play crucial roles in carcinogenesis, it is hard to anticipate the effect of ROS-modulating therapy via autophagy modulation and redox metabolism in cancer [11]. This review discusses how oxidative stress/damage and redox signaling govern autophagy in the context of cell survival or death. Additionally, it focuses on recent breakthroughs in our knowledge of how ROS formation, redox signaling, and oxidative stress change autophagy and the role of autophagy as a cell death or survival mechanism in response to oxidative stress in cancer.

## 2. Cellular Signaling and Physiological Roles of Redox Metabolism and Autophagy

The physiological functions of normal cells are conducted under redox equilibrium conditions, and additional ROS in normal cells cause tumors [12], although a redox imbalance causes oxidative stress. DNA damage is one of the earliest steps in tumorigenesis, involving malignant altered somatic cells [13]. Recently, excess ROS was found to cause oxidative DNA damage, genomic instability, and mutations [14]. This impact is mainly evident in proto-oncogenes and tumor suppressor genes, where unrepaired mutations can promote tumorigenesis [14]. However, a redox equilibrium is a physiological need in normal and cancer cells. Tumors are caused by redox conditions, while tumorigenesis and tumor formation entail ROS production and removal [15]. Therefore, disturbances in redox homeostasis contribute to cancer onset and progression.

**Figure 1:**
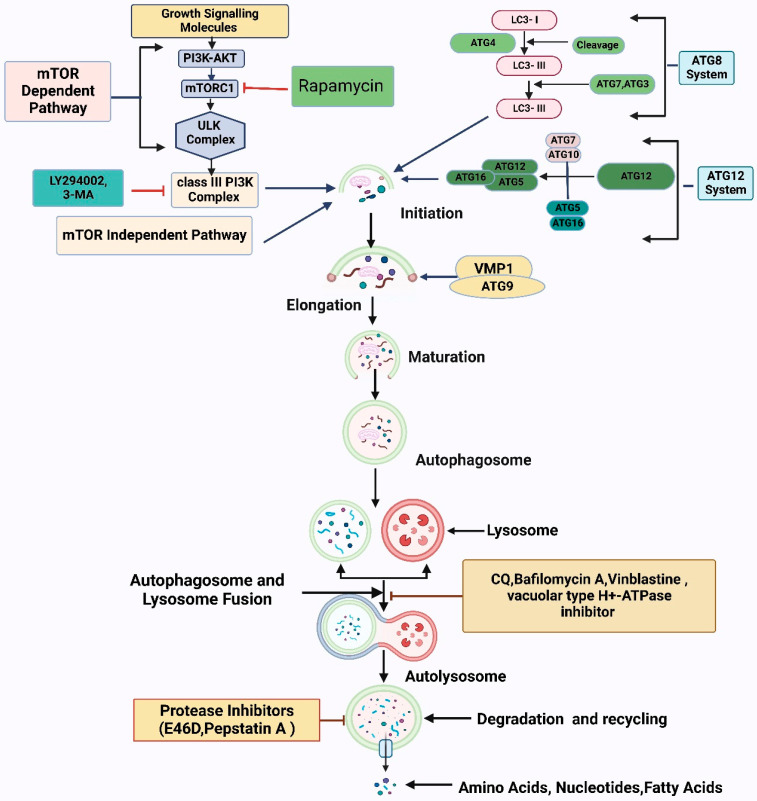
The autophagy pathway’s biological role and its underlying molecular mechanism. The autophagy process starts when the action of several proteins creates a structure called a pre-autophagosome. The interaction of the ULK1/VPS34/beclin-1 complex impacts phosphoinositide 3-kinase (PI3K)-AKT and the mammalian target of rapamycin (mTOR) to begin pre-autophagosome assembly. Additionally, the ATG5/ATG12/ATG16 and ATG12/ATG5/LC3 complexes are involved in causing phagophore nucleation and the accumulation of elongated macromolecules, in addition to their ability to bind to the developing autophagosomes. With the assistance of the ESCRT/SNARE/RAB7 protein complex, lysosomes can attach to mature autophagosomes, ultimately leading to autolysosome production. Finally, acid hydrolases successfully dismantle autolysosomes, liberating metabolites and nutrients.

Autophagy is controlled through signaling pathways, including autophagy-related genes (ATGs) [16,17]. Autophagy has five stages: initiation, phagophore nucleation, elongation, fusion, and degradation [5]. Autophagy begins when ULK1 (ATG1) is released from mammalian target of rapamycin (mTOR) inhibition [18]. ULK1, ULK2, FIP200, ATG101, and ATG13 lead to phagophore nucleation, which is triggered by a class III phosphoinositide 3-kinase (PI3K) complex containing VPS15, VPS34, ATG14, beclin 1, UVRAG, and AMBRA1 [19]. ULK1 phosphorylates beclin 1, a PI3K protein scaffold that speeds phagophore protein recruitment [20]. Autophagosome formation involves two ubiquitin-like conjugation mechanisms [21]. First, phosphatidylethanolamine (PE) binds to internal LC3-I to create LC3-II, the lipidated LC3 form; ATG4B, ATG3, and ATG7 accelerate this conversion, incorporating LC3-II into the developing double membrane [22]. ATG7 and ATG10 mediate the second system, which comprises ATG5-ATG12 [23]. Finally, syntaxin 17 (STX17) stimulates autophagosome–lysosome fusion and autophagosome destruction [24]. Autophagy’s detailed mechanism and regulation are presented in Figure 1.

ROS and reactive nitrogen species (RNS) are important intracellular signal transducers that are necessary for maintaining autophagy [25]. ROS generation and thiol redox imbalance are autophagy mediators caused by food restriction. ROS and RNS might change proteins at the level of sulfur-containing residues (cysteine and methionine), giving evidence of a redox-based signal [26]. ROS and RNS oxidize DNA and cellular macromolecules, causing biological harm [27]. However, the reactive cysteine thiol groups (SH) of many proteins may rapidly react with hydrogen peroxide (H_2_O_2_) and nitric oxide (NO) in biological systems, creating S-hydroxylated (S-OH) and S-nitrosylated (S-NO) derivatives, respectively [25]. Upon reacting with additional cysteines (e.g., glutathione or protein thiols), both adducts are converted to disulfide (S-S) and eventually reduced back to sulphhydryl at the expense of reduced nicotinamide adenine dinucleotide phosphate through the thioredoxin reductase or glutaredoxin reductase systems [28]. Oxidative alterations of reactive cysteines impact protein structure and function, localization, and post-translational modification. Reactive cysteines are the key redox-signaling molecular switches [29]. p62 was found to be related to autophagy and Nrf2 signaling. The redox-independent interaction between autophagy and the antioxidant response via the p62/Keap1/Nrf2 pathway is also thought to offer a broad perspective on autophagy and oxidative stress [30]. To prevent intervertebral disc degeneration, Nrf2 is responsible for driving oxidative-stress-induced autophagy in nucleus pulposus cells through a feedback loop involving Keap1/Nrf2/p62 [31]. Nrf2 protects cells from free radical stress mediated cell death. Recently, ubiquitin-binding protein p62-mediated autophagy has been shown to activate NRF2 and eliminate mitochondrial malfunction and oxidative stress [32]. Additionally, the Nrf2-Keap1-ARE pathway protects cells from oxidative stress, environmental toxins, and hazardous substances through cytoprotective genes.

Multiple regulatory systems provide evidence of a reciprocal relationship between autophagy and redox signaling. Cellular signaling and physiological evidence suggest that aberrant cell death and redox signaling contribute to cardiac, neurological, and metabolic disorders [33]. Cells use autophagy to control abnormal cell death and oxidative stress as a cytoprotective approach. Autophagy dysfunction can cause mitochondrial dysfunction and ROS [34]. The cellular machinery governing redox, autophagy, and aging has been identified. Redox signaling regulates autophagy and aging, altering the redox equilibrium [35]. Oxidative stress and autophagy are highly interactive. Increased ROS production was found to initiate and control autophagy [20]. However, autophagy regulates redox metabolism by removing damaged molecules or organelles. Additionally, mitophagy removes damaged mitochondria, an ROA source [36]. Mitophagy was found to protect mitochondrial integrity and oxidative equilibrium by lowering free radical production, although reduced mitophagy impairs mitochondrial breakdown, causing oxidative stress [37]. Meanwhile, mitochondrial dysfunction and elevated ROS are associated with cardiovascular disease, dementia, carcinogenesis, chronic inflammation, and cancer [38]. Moreover, mitophagy prevents cell death and tissue damage, and mitofusin 2 (MFN2) reduces angiotensin II-induced cardiomyocyte damage by lowering intracellular ROS generation [39]. Therefore, redox metabolism, ROS production, and autophagy interact to maintain cellular function and regulation.

## 3. Interplay between Redox Signaling and Autophagy in Cancer

**Figure 2:**
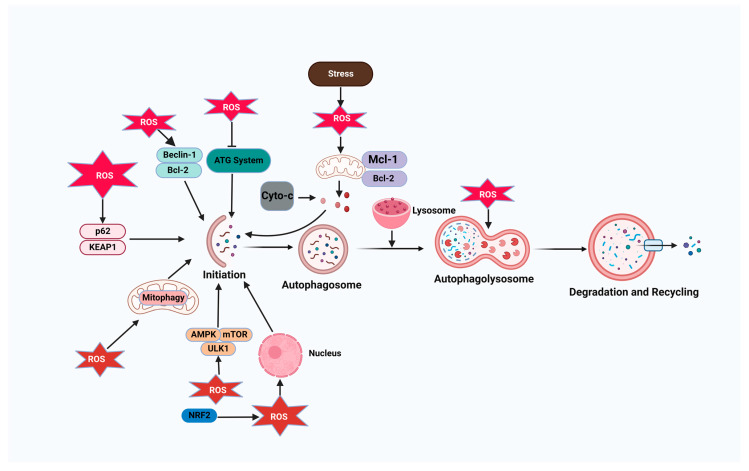
Autophagy–reactive oxygen species (ROS) relationship. ROS levels regulate autophagy through different pathways, including ATG4 oxidation, leading to autophagosome accumulation; the activation of the AMPK signaling cascade to induce autophagy through the ULK1 complex; the disruption of the beclin-1-Bcl2 interaction, leading to autophagy initiation; and the alteration of mitochondrial homeostasis, leading to mitophagy activation. Autophagy limits ROS accumulation by mitophagy or selective autophagy mediated by SQSTM1/p62 and NRF2-regulated antioxidant genes.

Autophagy removes oncogenic chemicals, toxic unfolded proteins, and defective organelles. However, once a tumor develops, elevated autophagic levels help cancer cells survive, proliferate, and migrate [40]. Cancer cell autophagy interacts with redox regulation. Oxidative stress causes cancer cell autophagy [41]. H_2_O_2_ can enhance LC3-PE accumulation by blocking ATG4’s cytoplasmic delipidating activity and triggering autophagy in amino-acid-starved cells [42]. Oxidative stress activates the transcription factor forkhead box O3 (FOXO3) in MCF-7 breast cancer cells, increasing the transcription of autophagy-related LC3 and BNIP3 [43]. Because cancer cells have hypermetabolic and dysfunctional mitochondria, their ROS levels are much greater than those of normal cells. Excess ROS are harmful to cancer cells [44]. Cancer cells continually strengthen their antioxidant defenses to eliminate excess ROS and survive under extreme oxidative stress [45]. Autophagy helps cancer cells tolerate oxidative damage, promoting tumor growth. High autophagic flux may prevent an increase in ROS generation in hypoxic cancer cells by promoting their survival. Hypoxia-inducible factor-1α (HIF-1α)-induced autophagy and mitophagy lower ROS levels in cancer cells under hypoxia [46]. Oxidative stress can trigger autophagy via AMPK. After H_2_O_2_ buildup, AMPK can be phosphorylated by AMPK kinase [47]. Activated AMPK phosphorylates and activates ULK1 to promote autophagy and inhibits mTORC1 by phosphorylating TSC2 and RAPTOR during glucose deprivation, reducing mTORC1’s inhibition of ULK1 [48]. The connection between autophagy and ROS levels governs autophagy in various ways (Figure 2). Therefore, a high autophagic flux eliminates ROS in cancer cells to prevent their harmful effects, enhancing tumor development.

Recently, it was found that ROS-induced autophagy mediates cell protection and that ROS regulates autophagy in cancer cells [29]. It was also found that AMPK-mTORC1-ULK1 maintains normal and malignant cell redox states. AMPK activation by ROS increases mitochondrial biogenesis and is proportionate to mitochondrial oxidative phosphorylation and ROS production [40]. Several studies have shown that the active Nrf2/antioxidant response element (ARE) pathway functions in mitochondrial biogenesis, acting with the transcriptional co-activator PGC1 to regulate electron transport chain components and enzyme production [49]. The interaction between the NRF2 and PGC1 signaling pathways in regulating mitochondrial biogenesis as a means of activating longevity highlights the role that NRF2 plays in mitochondrial biogenesis and its interaction with PGC1 in increasing longevity [50].

Under normal physiological conditions, Keap1, a cytosolic inhibitory protein, leads to Nrf2 proteasomal degradation, which is essential in regulating the Keap1-Nrf2 pathway and the autophagy process [51]. During oxidative stress, Keap1 inactivation leads to NRF2 activation. Upon counter-activation, Nrf2 enters the nucleus, dimerizes with Nrf1 or Maf, and interacts with AREs, inducing antioxidant machinery, such as glutathione-S-transferase (GST), to maintain redox homeostasis [52]. Additionally, long-term AFB1 therapy lowers JNK-mediated cell death in macrophages by activating cytoprotective autophagy through the interaction of UPR and GSTO1-1 [40]. Beclin-1 was downregulated in human prostate, colorectal, breast, and ovarian cancers, indicating the relevance of autophagy in tumor growth and survival to maintaining redox homeostasis [53]. New therapeutic targets include Nrf2 inducers and inhibitors. The Keap1-Nrf2 system’s molecular underpinnings must be targeted to improve translational research [54]. Recent studies found crosstalk between Nrf2 and other signaling pathways, highlighting how the Keap1-Nrf2 system regulates health and disease [55]. Therefore, autophagy modulation in cancer cells causes programmed cell death by dysregulating the redox balance.

## 4. Autophagy Controls ROS Production and Antioxidants to Regulate Cellular Redox Homeostasis

**Figure 3:**
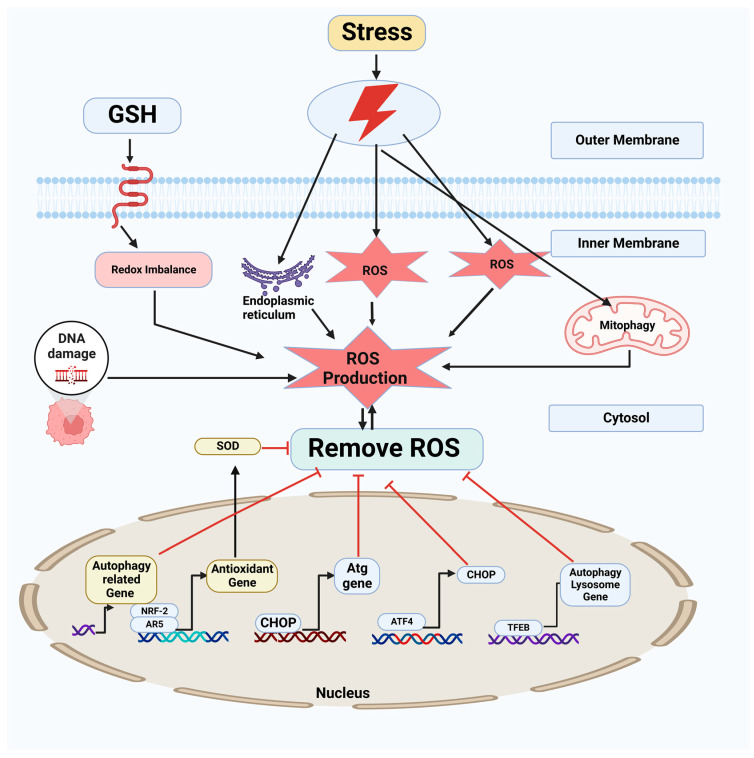
ROS homeostasis is regulated by autophagy. ROS facilitate autophagy by severing the connections of TFEB with RRAG GTPase and beclin 1 with Bcl2. ROS cause a conformational change in Keap1, ultimately resulting in the dissociation of the Keap1-Nrf2 complex, another mechanism through which ROS activate Nrf2. Therefore, cells can alleviate increased oxidative stress through the induction of autophagic activity and antioxidant proteins dependent on TFEB and Nrf2.

It has been suggested that oxidative stress may play a role in the demise of autophagic cells, which has been associated with several clinical disorders. Autophagic cell death involves the excessive breakdown of critical biological components [56]. Autophagy can also cause cell death by permeating lysosomal membranes in response to stress [57]. Cathepsins released from lysosomes mediate oxidative-stress-induced apoptosis [58]. Lysosomes, a source of iron and ROS, may exacerbate oxidative damage. In animal models and HeLa cells, autophagy was activated in starving cells, and starvation-induced autophagy requires mitochondrial superoxide (O_2_•^−^) production, activating AMPK [59]. In endothelial cells, 2-deoxy-D-glucose-induced autophagy is mediated by AMPK via ROS production [60]. Hypoxia–reoxygenation-induced ROS promote autophagy, whose inhibition enhances hepatocyte apoptosis [61]. Additionally, the nuclear factor (erythroid-derived 2)-like 2 transcription factor mediates the oxidative-stress-induced transcription of antioxidant genes through cis-acting sequences called AREs [62]. Keap1-Cul3 sequesters Nrf2 in the cytoplasm and degrades it through the ubiquitin-proteasome-dependent pathway. Oxidants or electrophiles modify Keap1’s Cys273 and Cys288 to prevent Nrf2 ubiquitination and nuclear translocation [63]. Atg7/p62-dependent Keap1 autophagy activates Nrf2 and reduces oxidative damage [64]. ROS affect autophagy indirectly and directly. ROS indirectly regulate autophagy via transcription and post-transcription. AMPK, beclin 1, PI3K, and other molecules are modified and interact with ROS to regulate autophagy post-transcriptionally, while p62, Keap1, and Nrf2 regulate autophagy transcriptionally. ROS can directly oxidize ATG4 and p62, inhibiting autophagy [65]. Moreover, several studies on antioxidant dietary supplements have provided inadequate evidence of antioxidants helping to prevent or inhibit cancer via autophagy. Combining antioxidants with radiochemotherapy has shown promise in cancer treatment [66]. In addition, a phase I clinical study of ascorbic acid with gemcitabine indicated good tolerance and tentative effectiveness in pancreatic cancer via autophagy modulation [67]. Another phase I clinical trial of ascorbate with radiation and temozolomide for newly diagnosed glioblastoma showed that their combination was safe and required further study [68]. Meanwhile, antioxidants may minimize adverse radiation effects. Recently, GC4419 (a superoxide dismutase mimic) was shown to decrease the severe oral mucositis caused by radiation and cisplatin in head and neck cancer patients [69]. When applied to tumors, pro-oxidant therapy was shown to work by increasing ROS levels and exacerbating oxidative stress in cancer cells to promote their deaths and inhibit tumor progression [70]. This effect is because cancer cells upregulate antioxidant production to eliminate excess ROS and maintain redox homeostasis (Figure 3) [61]. Therefore, these clinical findings require further research to validate the therapeutic benefit of combining antioxidants with chemoradiotherapy.

## 5. Recent Update and Therapeutic Application of Redox Homeostasis and Autophagy in Cancer

**Figure 4:**
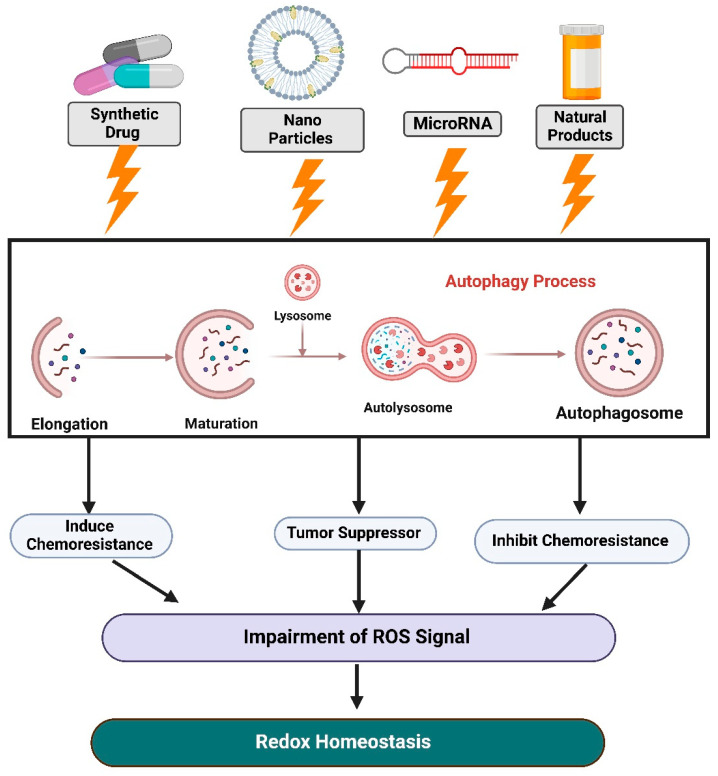
Schematic diagram of the latest findings on redox homeostasis and autophagy in cancer and their potential therapeutic applications. Targets for redox homeostasis and autophagy in cancer include synthetic drugs, natural chemicals, nanoparticles, and microRNA-mediated pathways.

Our knowledge of cancer metabolic redox pathways remains in its infancy, despite their potential to offer unique therapeutic opportunities for future cancer interventions. The creation of small molecules precisely targeting redox metabolism has the potential to be translated into effective cancer treatments via autophagy. Here, we separately describe the potential therapeutic applications of redox homeostasis and autophagy in cancer, as summarized in Figure 4.

### 5.1. Synthetic Drugs Targeting Redox Homeostasis and Autophagy in Cancer

Synthetic drugs targeting redox homeostasis and autophagy have recently been tested in patients with several types of cancer. Therefore, creating antioxidant-specific inhibitors and gaining a better understanding of the function of antioxidants in maintaining redox homeostasis are critical steps in treating cancer. Autophagy inhibitors, such as 3-methyladenine (3-MA), and autolysosome inhibitors, such as ammonium chloride (NH_4_Cl), bafilomycin A (BafA), and chloroquine, might reduce CLK4-mediated MITF degradation. It was found that 3-MA consistently inhibited MITF degradation. CLK4 enhanced MITF autophagy in esophageal squamous cell carcinoma [71]. A chelator for redox-active metal ions (KS10076) stimulated ROS-mediated STAT3 degradation in autophagic cell death and removed ALDH1^+^ stem cells [72]. A possible new use for metformin in treating photoaging is its ability to modulate autophagy, apoptosis, and oxidative stress. Metformin’s anti-photoaging effect is primarily related to its ability to enhance autophagic flux by activating cathepsin D. This effect is in addition to its antioxidant, anti-inflammatory, and antiapoptotic activities [73]. In vitro testing showed that exposure to FK866 made pancreatic ductal adenocarcinoma cells more susceptible to the antiproliferative effects of metformin and reduced nicotinamide adenine dinucleotide (NAD^+^) levels in cells. Intriguingly, combining FK866 with metformin enhanced survival in mice with KP4 cell line xenografts but did not have this effect in mice with PANC-1 cell line xenografts [74]. Biodegradable ferric phosphate nanosheets coated with doxorubicin facilitated tumor eradication via an autophagy-inhibition-enhanced apoptosis/ferroptosis pathway [75]. Nicotinamide (niacin) improved lipid metabolism and ROS-induced energy disruption in triple-negative breast cancer, suggesting medication repositioning as a means for providing additional antitumor agents [76]. Diclofenac (DCF) and cisplatin decreased *Bcl2*, *BclxL*, *cIAP1*, and cyclin D1 expression in KATO/DDP cells compared to cisplatin alone. Reduced mitogen-activated protein kinase (MAPK), Akt, NF-B, AP-1, and STAT3 activation contributed to this effect. DCF potentiated cisplatin’s anticancer activity in signet ring cell gastric carcinoma by regenerating intracellular ROS, promoting cell death via autophagy, and altering the cell survival signal transduction system [77]. Combining RAD001 with Rhein lowered tumor weight and volume, inhibited p-PI3K, p-Akt, and p-mTOR levels, and repressed *Ki-67* expression, exerting synergistic cancer prevention in gastric cancer in vivo. Rhein and RAD001 inhibit gastric cancer through PI3K/Akt/mTOR [78]. Rhein’s anticancer effect in pancreatic cancer cells grown in vitro is increased when hypoxia-induced HIF-1α-mediated autophagy is inhibited with an mTOR inhibitor [79]. Recently developed synthetic drugs that maintain redox homeostasis in autophagy-mediated signaling in a number of cancer cells are listed in Table 1.

### 5.2. Natural-Compound-Mediated Targeting of Redox Metabolism and Autophagy in Cancer

The medicinal compounds that are currently available for treating cancer that target redox regulation and autophagy are listed in Table 2. One study explored the anticancer effects of isoliquiritigenin in pancreatic cancer for the first time, including its roles in antioxidation, metabolic redox control, and autophagy [80]. Metformin suppressed the impact of low-dose resveratrol on tumor growth and enhanced the antitumor efficacy of high-dose resveratrol in triple-negative breast cancer by increasing its reducibility [81]. The effects of combining resveratrol with high-intensity interval training on the hippocampus of elderly male rats were used to examine mitochondrial signaling pathways [82]. A network pharmacology study investigated and experimentally confirmed curcumin-related mechanisms against hepatocellular carcinoma [83]. The antioxidant activity of polyphenols is thought to confer several positive effects. Fruits and vegetables such as grapes and tomatoes contain the polyphenol flavonoid kaempferol. Kaempferol increased phosphorylated AMPK, LC3-II, and beclin 1 levels in gastric cancer cells to trigger autophagy and cell death [84]. The health benefits and food industry applications of phytochemical compounds in various botanical parts of *Morus* species (e.g., oxyresveratrol) have been examined [85]. Quercetin activates AMPK and causes HIF-1α accumulation, repressing mTOR signaling and increasing the production of Bcl2/adenovirus E1B 19 kDa protein-interacting protein 3/ligand (BNIP3/BNIP3L) to disrupt the beclin 1/Bcl2/BclxL complex and induce autophagy [86]. The toxicity caused by honokiol microemulsion is stage-dependent and is caused by its dual roles in oxidation–reduction and apoptosis mediated by the FOXO autophagy signaling pathway [87]. When treated with ginsenoside, gastric cancer cells may undergo apoptosis, autophagy, and arrest in the cell cycle through ROS modulation and MAPK pathway activation [88]. Recently, in vitro and in vivo studies found that ginseng root extract can reduce inflammation by inhibiting the MAPK/NF-kB signaling pathway and stimulating autophagy and the p62-Nrf2-Keap1 signaling pathway [89]. Another study tested genistein in the SW480, SW620, and HaCaT cancer cell lines. Genistein is selective for the SW480 and SW620 cell lines. It suppresses nuclear receptor co-repressor (N-CoR) misfolding, activating the oncogenic survival pathway in non-smooth-cell lung cancer and is related to the autophagy molecular chaperone HSC70 [90]. A dose-dependent antiproliferative action reduces cell viability. Increased ROS production suggests an association with cell death through several pathways [91]. Apigenin induced apoptosis in cutaneous squamous cell carcinoma patients by downregulating the expression of sulfiredoxin by inducing apoptosis [92]. A recent paper reviewed the intricate association between autophagy and ROS in cancer, phytochemicals regulating ROS and autophagy for cancer treatment, ROS/autophagy inhibitors’ effects on phytochemical anticancer properties, and the challenges of using phytochemicals to regulate ROS and autophagy for cancer treatment [93]. S-Adenosylmethionine (AdoMet), a natural chemical and nutritional supplement, is known for its antiproliferative and pro-apoptotic properties in several human malignancies. AdoMet stimulates ER stress, autophagy, miR-888-5p downregulation, and MYCBP and CDH1 upregulation in laryngeal squamous cancer cells (LSCC), making it a promising miRNA-mediated cancer treatment and prevention method [94]. However, it is required to describe the molecular processes responsible for the redox regulation of autophagy in cancer in detail and then explain the ROS- and autophagy-based therapeutic methods that are used to treat cancer [65].

### 5.3. Nanoparticle-Mediated Targeting of Redox Metabolism and Autophagy in Cancer

Nanoparticles (NPs) can be used in cancer diagnostics and treatment. A recent paper discussed the typical procedures that are used to prepare NPs. Then, an in-depth analysis was conducted of how many different protein NPs may be used to improve cancer imaging and treatment [95]. Various cancer cells often use silver NPs (AgNPs) to maintain the redox balance. AgNPs have noticeable neurotoxic effects on SH-SY5Y cells, as indicated by reduced *APP* and *ADAM10* gene expression, suppressed cell proliferation, and elevated BACE1 protein levels. AgNPs cause oxidative stress and size-dependent neurotoxicity in SH-SY5Y neuroblastoma cells [96]. The biocompatibility, simple and controlled production, strong anticancer activity, and photothermal conversion capability of gold NPs (AuNPs) have all been considered when choosing these materials [97]. A zinc oxide (ZnO) NP treatment decreased P13K/AKT/mTOR signaling in MG63 cells. ZnO NPs triggered apoptosis and autophagy in MG63 cells by altering associated proteins [98]. In RAW264.7 cells, magnetic iron oxide NPs caused autophagy before cell death by damaging mitochondria and the endoplasmic reticulum [99]. An innovative smart PEGylated gelatin NP co-delivering doxorubicin and betanin increased chemotherapy’s therapeutic effectiveness [100]. Both redox metabolism and autophagy have been suggested as potential NP cancer targets in the various cells listed in Table 3.

### 5.4. MicroRNA-Mediated Targeting of Redox Metabolism and Autophagy in Cancer

Recently, redox metabolism and autophagy were identified as cancer processes that could be targeted using microRNAs (miRNAs). MiRNAs are key redox regulators of chemo/radio-resistance (Table 4). MiRNAs regulate antioxidant enzymes, redox-sensitive signaling pathways, cancer stem cells, DNA repair, and autophagy to regulate treatment resistance [101]. The tumor suppressor miRNA-133a-3p is known to prevent autophagy-mediated glutaminolysis, further inhibiting gastric cancer development and spread [102]. It was also found that miRNA-7 inhibited glucose pools generated by autophagy, inhibiting pancreatic cancer growth [103]. Targeting ULK2 with miRNA-26b inhibited autophagy in prostate cancer cells [104]. In hepatocellular carcinoma, glycine decarboxylase is responsible for inducing autophagy and is suppressed by miRNA-30d-5p [105]. miRNA-335-5p stimulates AMPK, which is involved in mTOR signaling and autophagy [106]. MiRNA-335-5p activated autophagy to reduce OA chondrocyte inflammation. MiRNA-93 reduced TNF-, IL-1-, and IL-6-induced chondrocyte inflammation [107]. By targeting SIRT1, miRNA-494 can inhibit hypoxia/reoxygenation-induced cardiomyocyte apoptosis and autophagy through the PI3K/AKT/mTOR signaling pathway [108]. It is believed that miRNA-486-5p downregulates PTEN, activating the PI3K/Akt signaling pathway and inhibiting autophagy activity in MCF-7 breast cancer cells [109]. MiRNA-489-mediated tumor suppression and chemosensitization suggest that miRNA-489 could be used as a therapeutic sensitizer in a specific subgroup of patients with treatment-resistant breast cancer [110]. Long noncoding RNAs (lncRNAs) and miRNAs can influence autophagy, epithelial–mesenchymal transition (EMT), and their interplay by modulating molecular signaling pathways. Autophagy- and EMT-related lncRNAs and miRNAs may aid cancer diagnosis, prognosis, and treatment [111]. A weighted gene co-expression network analysis served as the foundation for constructing and subsequently experimentally confirming ferroptosis-related competing endogenous RNA networks in hepatocellular cancer [112]. The miRNA 149-5p plays both a tumor-suppressing and an oncogenic role in human cancers [113]. Therefore, autophagy-regulating miRNAs may determine whether it generally promotes or represses tumorigenicity to modulate redox metabolism in cancer.

## 6. Limitations and Future Perspectives on Redox Metabolism and Autophagy in Cancer

Because autophagy plays roles in interactions between tumors and their hosts and the development of tumor immunity, it is imperative that immunocompetent animal models be the primary focus of autophagy research. There has been some progress in understanding the function autophagy plays in maintaining stem-like cancer cells and metastases [116], but there remains a lot more to learn. While we understand cancer at the redox level, malignancies are complicated illnesses involving various variables and pathways. Excess ROS damage normal cells. ROS and antioxidants may regulate immunological function in tumors [117]. Recently, it has been found that natural chemicals decrease cancer cell growth, invasion, angiogenesis, and metastasis [118]. Additionally, antioxidants from natural phytochemicals can operate as crucial signaling molecules [119], but their potential as phyto-chemotherapeutic agents remains unclear. Moreover, single-nucleotide polymorphisms, which are genetic variations, can also change how biologically active phytochemicals are, either positively or negatively, depending on the presence of chemopreventive compounds or hazardous environmental factors [120]. Some synthetic drugs targeting redox signaling pathways may pass through or be impacted by other pathways, reducing their anticancer effects [121], potentially with additional side effects. Moreover, NP-linked targeting improves phytochemical efficiency. NP toxicity prevents oral intake [122]. Therefore, biodegradable and eco-friendly NPs are increasingly becoming the NPs of choice in fighting cancer [123]. Alternatively, the effects of metabolic alterations on tumor stroma and redox metabolism and the effects of ROS on tumor growth are also noteworthy [124]. Respiration, glucose, and glutamine metabolism are all involved in ROS production and elimination [125]. It is possible that the most successful course of action would be to identify other potential synthetic lethal techniques, merging our expanding understanding of cancer genetics with autophagy suppression. Meanwhile, it is abundantly apparent that additional autophagy inhibitors that are more effective and selective are required, both as chemical tools and as clinical therapeutic candidates targeting redox signaling pathways. Therefore, further studies and better knowledge of malignancies will expose their secrets and enhance treatment techniques to aid redox metabolism and autophagy modulation in cancer. Therefore, much work remains before etiology or treatment concerns can be solved to enable redox-metabolism-mediated autophagy modulation in cancer in the future.

## 7. Conclusions

Autophagy and its mechanism regulate redox homeostasis and ROS production [126]. ROS sources include autolysosomes [127]. Redox signaling includes targeted alteration by a reactive species through a chemically reversible process without a pro-oxidant/antioxidant imbalance [128]. However, while different signaling events governing autophagy and how ROS/RNS activate/regulate signaling cascades are well understood, autophagy regulation in redox homeostasis remains to be understood. While autophagy plays a complicated role in cancer, inhibiting it may be useful in advanced cancer [129]. However, understanding tumor autophagy has led to new inhibitors and therapeutic trial methods to regulate redox homeostasis [130]. Identifying the patients most likely to benefit from this strategy still presents several challenges and possibilities. Finally, we should remember that cancer is not a single disease but hundreds of diseases. For example, the metabolic processes of melanoma and liver tumors are as different from one another as the form or function of the tissues that they arise from. It is quite conceivable that significant breakthroughs are on the horizon in the near future as a result of the extensive research efforts being put forth in this fascinating and novel but also very old topic. Therefore, additional synthetic drug target, natural compound, NP-mediated, and miRNA-based research optimizing doses and targets will generate both therapeutic and destructive effects on cancer cells to minimize redox homeostasis through autophagy modulation.

## Figures and Tables

**Table 1:** Synthetic drugs that were recently used to maintain redox homeostasis via autophagy-mediated signaling in several cancer cells.

Synthetic Drug	Model	Redox Mechanism	Autophagy Modulation	Reference
Ammonium chloride (NH_4_Cl), bafilomycin A (BafA), 3-methyladenine (3-MA), and chloroquine	Esophageal squamous cell carcinoma	Modulating redox status and nucleotide metabolism	Inhibition	[71]
KS10076	Cancer stem cells	Induces ROS-mediated STAT3 degradation	Induction	[72]
FK866	Pancreatic Cancer Cells	Decreasing oxidized (NAD^+^) to reduced (NADH) nicotinamide adenine dinucleotide ratio	Autophagy-mediated cell death	[74]
Doxorubicin	Tumor cells	Apoptosis/ferroptosis pathway	Impaired autophagy	[75]
Nicotinamide	Triple-negative breast cancer	Mitochondrial dysfunction and ROS activation	Autophagy modulation	[76]
Metformin	Ultraviolet (UVA)-exposed mice	Elevated oxidative stress	Enhanced autophagic flux	[73]
Diclofenac (DCF)	Cisplatin-resistant signet ring cell gastric carcinoma cells (KATO/DDP)	Reduction in antioxidant enzyme expression while inhibiting Nrf2 activity	Activation	[77]
Rapamycin	MiaPaCa-2 and PANC-1 pancreatic cancer cells	Inhibition of HIF-1α-mediated autophagy	Enhanced autophagy	[79]
Everolimus (RAD001)	Human gastric cancer cells (MGC-803)	Phosphorylation of PI3K/Akt/mTOR	Autophagy induction	[78]

**Table 2:** Recent use of natural compounds to maintain redox equilibrium in autophagy-mediated signaling in various cancer cells.

Natural Product	Model	Redox Mechanism	Autophagy Modulation	Reference
Isoliquiritigenin	Pancreatic cancer	ROS-autophagic redox homeostasis	Autophagy modulation	[80]
Metformin	Triple-negative breast cancer	Increased catalase activity and NAD(P)H level	Induction	[81]
Curcumin	Hepatocellular carcinoma	Promoted apoptosis via the p53 pathway	Autophagy induction	[83]
Resveratrol	Albino Wistar rats	Increased NAD^+^/NADH, SOD2, and AMPK levels	Autophagy modulation	[82]
Honokiol	Neuroblastoma cells	Oxidation–reduction	Autophagy induction	[87]
Ginsenoside	BGC-823 human gastric cancer cell line	Modulates ROS and MAPK	Activation	[88]
Oxyresveratrol	Several cancer models	Increased lysosomal activity	Autophagy induction	[85]
Genistein	Colorectal cancer	Increased ROS production	Autophagy modulation	[91]
Apigenin	Cutaneous squamous cell carcinoma patients	Maintained redox	Autophagy modulation	[92]
Ginseng root extract	RAW264.7 cells	MAPK/NF-kB signaling	Activation	[89]

**Table 3:** Redox metabolism and autophagy as cancer targets for nanoparticle-mediated targeting in different cells.

Nanoparticle	Model	Redox Mechanism	Autophagic Condition	Reference
Silver	Human neuroblastoma cancer cell line (SH-SY5Y)	Cellular redox homeostasis	Autophagy inhibition	[96]
Gold	Bulk cancer cells and cancer stem cells in breast carcinoma	Redox homeostasis	Autophagy modulation	[97]
Zinc oxide	MG63 human osteosarcoma cells	Suppressed P13K/AKT/mTOR signaling	Autophagy induction	[98]
Magnetic iron oxide	RAW264.7 cells	ER stress homeostasis	Autophagy induction	[99]
PEGylated gelatin	MCF-7 cells	Targeting sequestosome 1 (SQSTM1) and cathepsin F	Autophagy induction	[100]

**Table 4:** Targeting redox metabolism and autophagy in different cancer cells through microRNAs.

MicroRNAs	Model	Redox Mechanism	Autophagy Modulation	Reference
microRNA (miRNA) 133a-3p	Patient-derived xenograft model and human gastric cancer organoid model	Glutaminolysis	Autophagy-mediated	[102]
miRNA-7	Pancreatic cancer	Reprogrammed metabolic homeostasis	Autophagy modulation	[103]
miRNA-26b	Prostate cancer cells	ULK2 is a direct target of miRNA-26b	Autophagy inhibition	[104]
miRNA-30d-5p	Hepatocellular carcinoma	Decreased ROS-mediated ubiquitination of cofilin	Autophagy inhibition	[105]
miRNA-335-5p	Human osteoarthritis chondrocytes	Anti-inflammatory	Autophagy induction	[106]
miRNA-93	Retinal ganglion cells	AKT/mTOR	Autophagy induction	[114]
miRNA-20a-5p	Human hepatocellular cancer cells	Downregulated hypertrophic cardiomyopathy	Autophagy modulation	[115]
miRNA-494	Acute myocardial infarction	PI3K/AKT/mTOR signaling	Autophagy induction	[108]
miRNA-486-5p	MCF-7 breast cancer cells	Modulation of miRNA expression profile	Autophagy induction	[109]
miRNA-489	Breast cancer cells	ROS regulation	Autophagy induction	[110]
Long noncoding RNAs	Several cancers	Maintained redox balance	Autophagy modulation	[111]
Competing endogenous RNAs	Hepatocellular carcinoma	Ferroptosis-related competing endogenous RNA	Autophagy regulation	[112]
miRNA-149-5p networks	Human cancers	Lipogenesis and vascular endothelial cells	Autophagy modulation	[113]
